# Ionic-liquid-based approaches to improve biopharmaceuticals downstream processing and formulation

**DOI:** 10.3389/fbioe.2023.1037436

**Published:** 2023-02-07

**Authors:** Catarina Almeida, Augusto Q. Pedro, Ana P. M. Tavares, Márcia C. Neves, Mara G. Freire

**Affiliations:** CICECO-Aveiro Institute of Materials, Department of Chemistry, University of Aveiro, Aveiro, Portugal

**Keywords:** biopharmaceuticals, ionic liquids, biomanufacturing, downstream processing, purification platforms, formulation, ionic liquid-based approaches, biopharmaceuticals administration

## Abstract

The emergence of biopharmaceuticals, including proteins, nucleic acids, peptides, and vaccines, revolutionized the medical field, contributing to significant advances in the prophylaxis and treatment of chronic and life-threatening diseases. However, biopharmaceuticals manufacturing involves a set of complex upstream and downstream processes, which considerably impact their cost. In particular, despite the efforts made in the last decades to improve the existing technologies, downstream processing still accounts for more than 80% of the total biopharmaceutical production cost. On the other hand, the formulation of biological products must ensure they maintain their therapeutic performance and long-term stability, while preserving their physical and chemical structure. Ionic-liquid (IL)-based approaches arose as a promise alternative, showing the potential to be used in downstream processing to provide increased purity and recovery yield, as well as excipients for the development of stable biopharmaceutical formulations. This manuscript reviews the most important progress achieved in both fields. The work developed is critically discussed and complemented with a SWOT analysis.

## Introduction

Due to the massive growth and aging of global population and to the high incidence of autoimmune and chronic diseases, there is a huge demand for effective and safe drugs (Kesik‐Brodacka, 2018; Tavares et al., 2019; O’Flaherty et al., 2020). The pharmaceutical industry is majorly governed by traditional low molecular weight and synthetic pharmaceuticals, which represent more than 90% of all drugs currently available (Ferreira et al., 2016; Tavares et al., 2019). However, a relatively new class of therapeutic molecules, known as biopharmaceuticals, has been gaining increasing traction. Biopharmaceuticals, also known as biologics or biologic drugs, were first introduced in the 1980s and refer to all pharmaceutical products with therapeutic activity that are derived from biological sources (Chen et al., 2018; Hong et al., 2018; US Food and Drug Admnistration, 2018; Tavares et al., 2019; O’Flaherty et al., 2020; Pereira et al., 2021). They represent an important fast-growing sector within the global pharmaceutical industry, constituting *ca.* one-third of all drugs currently under development (Moorkens et al., 2017; Tavares et al., 2019; Pereira et al., 2021).

The manufacturing of biopharmaceuticals, also known as biomanufacturing, comprises two main stages—the upstream and downstream processing (Jozala et al., 2016; Hong et al., 2018; Tavares et al., 2019). The upstream processing encompasses several events responsible to promote the growth of the host cell that will further express and produce the biomolecule of interest. Afterwards, during the downstream processing, the target biomolecule is isolated and purified (Jozala et al., 2016). Biomanufacturing itself demands a multistep approach, in which precise quality control and accurate monitoring must be carried out to assure some criteria, including the biomolecule’s identity/quality, purity and stability (Tavares et al., 2019; Pereira et al., 2021). Purification strategies are currently dominated by chromatographic techniques, which are needed to efficiently remove all cell-, process- and product-associated impurities, while being of easy scale-up, reliable, and highly robust (Jozala et al., 2016). However, their main disadvantage is their high cost (Jozala et al., 2016; Castro et al., 2019; Tavares et al., 2019). It is, therefore, crucial to either develop novel cost-effective purification platforms or to find new ways to improve existent workflows, providing a high-quality and high-purity product, while simultaneously meeting regulatory requirements (Castro et al., 2019; Pereira et al., 2021).

**FIGURE 1:**
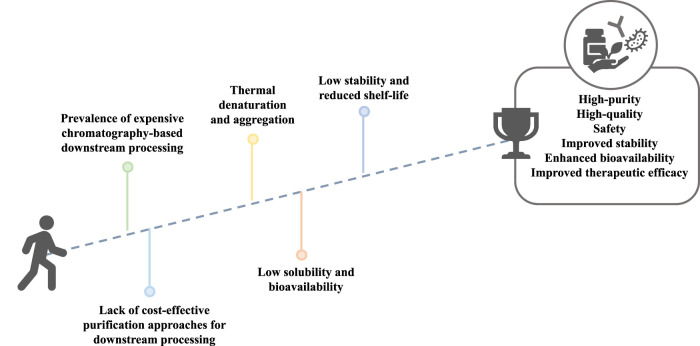
Current challenges faced in biomanufacturing.

Contrarily to synthetic drugs, due to their complexity and natural origin, biopharmaceuticals are often unstable (Lin et al., 2021; Loureiro et al., 2022). Thus, biopharmaceuticals formulation must ensure their long-term stability through the preservation of their structural integrity and bioactivity (Loureiro et al., 2022). This can be achieved by adding excipients to the final formulation or by employing costly preservation techniques that improve shelf-life and durability (Lin et al., 2021). Another important bottleneck is their low bioavailability *in vivo* after the administration, which can significantly alter pharmacokinetic properties and therapeutic efficacy (Lin et al., 2021; Loureiro et al., 2022). Altogether, these issues compromise the development of high-quality, effective, and safe biopharmaceuticals, which are major requirements in the sector (Tavares et al., 2019). The major challenges currently faced in the biopharmaceutical field are summarized in Figure 1.

ILs are salts formed by the combination of organic cations and organic or inorganic anions, resulting in salts with low charge distribution and low melting points, some of them liquid at room temperature, and capable of establishing a plethora of interactions (Pereira et al., 2021; Castro et al., 2019; e Silva et al., 2017). IL’s outstanding properties make them attractive for catalysis, electrochemistry, separation, and extraction, among others (Fontanals et al., 2009; Kianfar and Mafi, 2020). Although less investigated, within the pharmaceutical field, ILs have already shown to be important enhancers of the solubility, activity, permeability, and stability of several synthetic drugs (Pedro et al., 2020) and biopharmaceuticals (Vijayaraghavan et al., 2010; Byrne et al., 2012; Foureau et al., 2012; Kumar and Venkatesu, 2013; Mukesh et al., 2013; Kumar and Venkatesu, 2014; Mazid et al., 2015; Todinova et al., 2016; Banerjee et al., 2018; Jagannath et al., 2018; Pedro et al., 2018; Reslan et al., 2018; Guncheva et al., 2019; Lin et al., 2019; Quental et al., 2019; Mandal et al., 2020; Lin et al., 2021; Dhiman et al., 2022; Vicente et al., 2022), and also in the extraction and purification of these biomolecules (Shu et al., 2010; Qu et al., 2012; Yuan et al., 2012; Chen et al., 2015; Ding et al., 2015; Ren et al., 2015; Taha et al., 2015; Ferreira et al., 2016; Mondal et al., 2016; Ramalho et al., 2018; Santos et al., 2018; Song et al., 2018; Xu et al., 2018; Capela et al., 2019; Magri et al., 2019; Marchel et al., 2019; Quental et al., 2019; Vicente et al., 2019; Castro et al., 2020; Neves et al., 2020; Vicente et al., 2022; Capela et al., 2023).

## Biopharmaceuticals manufacturing

Most biopharmaceuticals are obtained through recombinant deoxyribonucleic acid (DNA) technology, in which a given gene is introduced in the genetic code of the host organism to produce the biomolecule of interest. Innovations have been achieved in this field, allowing to shift from production approaches based on non-mammalian systems to mammalian cell cultures (Tavares et al., 2019; Rasmussen et al., 2021). Within biopharmaceuticals are included vaccines, nucleic acids, cell or gene therapies, plant extracts, allergens, cytokines, tissue growth factors, blood components or derivatives, peptides, enzymes and recombinant therapeutic proteins, including monoclonal antibodies (mAbs) (Rosa et al., 2010; Foldvari et al., 2016; Chen et al., 2018; Tavares et al., 2019; O’Flaherty et al., 2020). The oldest biopharmaceutical approved by the U.S Food and Drug Administration (FDA) regulatory agency—a recombinant DNA human insulin produced in *Escherichia coli*—dates back to 1982 (The, 1989; O’Flaherty et al., 2020). Between 2013 and 2016, a total of 73 biopharmaceuticals were approved to be used in humans (Jozala et al., 2016). In 2016, this number increased, with the introduction of approximately 200 biopharmaceuticals in the market (Rasmussen et al., 2021). Later, in 2018, *ca*. 374 biopharmaceuticals obtained approval for therapeutic purposes in the European Union (EU) and the United States of America (United States of America) for the treatment of cancer, infectious and autoimmune diseases, and HIV-AIDS-associated disorders (Foldvari et al., 2016; Walsh, 2018; Parker and Li, 2021). Overall, the global market associated with biopharmaceuticals accounted for U.S. $237.2 billion in 2018 and it is expected to increase to U.S. $389 billion by 2024, with a compound annual growth rate (CAGR) of 8.59% (O’Flaherty et al., 2020). Antibodies correspond to the largest fraction of the biopharmaceutical market, in which monoclonal antibodies (mAbs) accounted for the highest sale numbers, with projections pointing out a market value of U.S. $200 billion by 2023 (Hong et al., 2018; Kesik‐Brodacka, 2018; Grilo and Mantalaris, 2019; O’Flaherty et al., 2020). Although mAbs currently represent the largest segment of the biopharmaceutical sector (Jozala et al., 2016; Castro et al., 2019; Tavares et al., 2019; Castro et al., 2020), ground-breaking research for the development of safe and effective delivery platform technologies to deliver nucleic acids has been at the forefront of the global efforts to fight the COVID-19 pandemic. Nucleic acids are gaining momentum within the biopharmaceutical sector, from which an ever-growing number of different products have received regulatory approval (Kulkarni et al., 2021).

**FIGURE 2:**
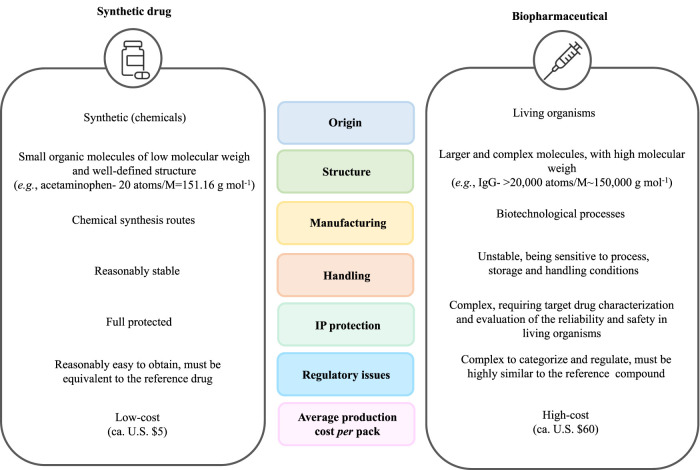
Comparison between conventional synthetic drugs and biopharmaceuticals.

Due to their safety, high specificity, and efficiency to tackle numerous diseases, in which a targeted therapy is provided instead of symptomatic therapy, biopharmaceuticals are gradually replacing synthetic therapies (Rosa et al., 2010; Kesik‐Brodacka, 2018; Tavares et al., 2019; O’Flaherty et al., 2020). Biopharmaceuticals significantly differ from the common synthetic drugs, namely concerning their origin, structure, manufacturing process, final formulation, handling conditions, dosing, immunogenicity, intellectual property (IP) issues, regulations and marketing, and cost, as summarized in Figure 2 (Rader, 2008; Kesik‐Brodacka, 2018; Tavares et al., 2019).

**FIGURE 3:**
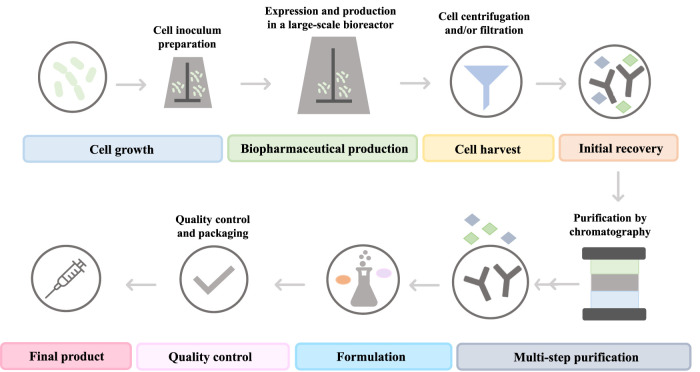
Representative overview of the general biomanufacturing of mAbs.

In contrast to biopharmaceuticals, which possess a biological nature, synthetic drugs are products resulting from chemical synthetic routes (Kesik‐Brodacka, 2018; Tavares et al., 2019). The latter are generally small organic molecules with low molecular weight and a simple and well-identified structure (e.g., acetaminophen has 20 atoms and a molecular weight of 151.16 g mol^−1^) (Information NC for B, 2007; Kesik‐Brodacka, 2018; Tavares et al., 2019; Rasmussen et al., 2021). In contrast, biopharmaceuticals are compounds of high molecular mass, being 200 to 1000 times larger than conventional drugs (e.g., IgG has more than 20,000 atoms and a molecular weight of *ca.* 150,000 g mol^−1^) (Tsang and Cortez, 2010; Mitragotri et al., 2014; Kesik‐Brodacka, 2018; Tavares et al., 2019; Rasmussen et al., 2021). Moreover, whereas synthetic drugs are characterized through well-established analytical techniques, the accurate characterization of biopharmaceuticals is more challenging. For instance, recombinant proteins, such as fragment crystallizable (Fc)-fusion proteins and mAbs, can undergo several post-translational modifications (proteolysis, glycosylation, among others), making their accurate bioprocessing and, consequently, their characterization, difficult to achieve (Jenkins et al., 2008). Moreover, biopharmaceuticals are more unstable and sensitive to the conditions employed during the manufacturing process, storage, and handling, such as temperature and pH (Kesik‐Brodacka, 2018; Tavares et al., 2019; Lin et al., 2021). Since biopharmaceuticals are highly prone to denaturation and degradation events, as well as to modifications in their amino acid and sugar patterns, the manufacturing conditions must be strictly controlled (Kesik‐Brodacka, 2018; Lin et al., 2021). As such, well-designed stabilization systems/excipients for these biomolecules are required to guarantee their long-term stability and preservation. Notwithstanding their outstanding properties, the IP protection and the regulatory approval pathways for biopharmaceuticals are more complex due to their manufacturing processes, since the characterization of the target drug and the evaluation of the reproducibility and safety of the production approach are mandatory (Tavares et al., 2019). Moreover, biopharmaceuticals are more expensive to produce (average production cost per pack was *ca.* U.S. $60 for biopharmaceuticals, in contrast with U.S. $5 per pack of small-molecule drugs) (Makurvet, 2021). A representative overview of the biomanufacturing of mAbs, including all steps required in the upstream and downstream stages, is provided in Figure 3. The upstream processing starts with the cell culture in optimal conditions designed to assure the proper cell growth in the culture media (Jozala et al., 2016; Hong et al., 2018). This process is then scaled up through a sequence of various bioreactor stages; the obtained cell mass is then transferred to the final bioreactor to allow the expression and production of the target biomolecule (Shukla and Thömmes, 2010; Hong et al., 2018). Afterwards, cells and cell debris are separated from the culture broth by a sequence of cell harvesting steps, involving centrifugation and/or filtration approaches, to obtain a clarified broth (Shukla and Thömmes, 2010; Jozala et al., 2016; Hong et al., 2018). The next stage of the manufacturing of biopharmaceuticals relies on downstream processing, during which, through a series of multiple steps, the biopharmaceutical is isolated and purified (Conner et al., 2014; Jozala et al., 2016; Hong et al., 2018; Tavares et al., 2019). The initial recovery of the target compound depends on the cellular localization of the biomolecule; for instance in the cases where the target compound is produced extracellularly, the clarified broth is concentrated by ultrafiltration and then purified, whereas when it is produced intracellularly, the cell is subjected to lysis after harvesting, and then clarified to remove the resultant cell debris (Jozala et al., 2016). The second step in the downstream processing corresponds to the purification of the target biomolecule, during which all impurities related to the cell (e.g., DNA and host cell proteins), the process (e.g., antifoam agents, leached ligands, and buffers), or the impurities associated to the product (fragments, clipped species, and aggregates) are removed (Azevedo et al., 2009; Conner et al., 2014; Rathore and Kapoor, 2015; Jozala et al., 2016). During this step, several techniques can be employed, such as chromatography, filtration, diafiltration, tangential flow filtration, as well as the use of aqueous biphasic systems (ABS) (Azevedo et al., 2009; Conner et al., 2014; Jozala et al., 2016; Hong et al., 2018; Tavares et al., 2019). The selection of the purification technique is also dependent on the cellular localization of the biopharmaceutical; for instance, if it is extracellularly produced, it can be purified by ultrafiltration, precipitation, and chromatography, whereas if it is intracellularly produced, it can be purified by precipitation and chromatography after cell lysis (Jozala et al., 2016).

The final goal of biomanufacturing is to obtain a safe and high-quality biopharmaceutical with high purity, high yield, low-cost, and within the shortest time (Jozala et al., 2016). Downstream strategies are currently dominated by liquid chromatography due to its high selectivity and high resolution (Azevedo et al., 2009; Fields et al., 2016; Jozala et al., 2016; Castro et al., 2019). Chromatographic techniques include affinity chromatography, which is based on the selective molecular recognition between an affinity ligand and the target molecule; reverse-phase and hydrophobic interaction chromatography, which separate molecules according to their hydrophobicity; size-exclusion and ion-exchange chromatography, in which the separation is achieved according to the molecular size and charge, respectively; and mixed-mode chromatography, which is a combination of the previously mentioned approaches (Saraswat et al., 2013; Hanke and Ottens, 2014; Jozala et al., 2016; Tavares et al., 2019). For example, the purification of mAbs generally requires a sequence of three chromatographic steps (Shukla et al., 2007; Azevedo et al., 2009). The mAbs capture step employs Protein A affinity chromatography, a technique based on the specific interaction between the mAbs Fc region and the Protein A ligand—a recombinant protein derived from *Staphylococcus aureus*—that is immobilized in a resin support (Shukla et al., 2007). Afterwards, two more chromatographic steps are required to polish the final product by removing impurities, such as aggregates of high molecular mass, host cell proteins, clipped species of low molecular mass, DNA, and leached Protein A.

Despite the high selectivity, high resolution and high purity of the product, chromatography also exhibits some limitations, namely low associated throughputs, low proteolytic and chemical stabilities when biological ligands are used, high scale pressure drops, time-consuming nature, and high costs, which hinder the production of biopharmaceuticals at low cost (Rosa et al., 2010; Jozala et al., 2016; Castro et al., 2019; Castro et al., 2020). To surpass these limitations, research has focused on the development of chromatographic ligands with enhanced selectivity, robustness, and reproducibility (Martins et al., 2014), as well as on non-chromatographic strategies, such as affinity precipitation (Hoffman et al., 2001; Roque et al., 2007), preparative electrophoresis (Thomas et al., 2002), high-performance tangential flow filtration (Lazarova et al., 2001), membrane filtration (Castilho et al., 2002), precipitation (dos Santos et al., 2017), aqueous biphasic systems (Zijlstra et al., 1998; Azevedo et al., 2009), non-chromatographic magnetic separation (Karl and Schwa, 2005), high-gradient magnetic separation (Akgo et al., 2004; Gomes et al., 2018), and crystallization (dos Santos et al., 2017). Nevertheless, the progress made in this field is still far from that required, and downstream processing continues to account for the most expensive part of biomanufacturing (Azevedo et al., 2009). More than 80% of the total production costs of a biopharmaceutical are allocated to downstream processing, in which approximately 20% results from the recovery, isolation, and polishing steps, and more than 70% from the purification step (Azevedo et al., 2009). After the final polishing stage, filling and finishing steps are applied, in which the biopharmaceutical is placed in the final formulation (Azevedo et al., 2009; Rosa et al., 2010; Conner et al., 2014; Jozala et al., 2016; Castro et al., 2021). Conventional approaches to enhance and/or maintain the stability of therapeutic biomolecules are focused on the addition of several compounds, acting as excipients, such as sugars, salts, amino acids, surfactants and polymers (Frokjaer and Otzen, 2005; Kerwin, 2008; Qian et al., 2008; Wang, 2015; Castro et al., 2021; Lin et al., 2021). These allow to prevent their denaturation and increase their bioactivity and shelf-life.

## Ionic-liquid-based approaches in the biopharmaceuticals field

**FIGURE 4:**
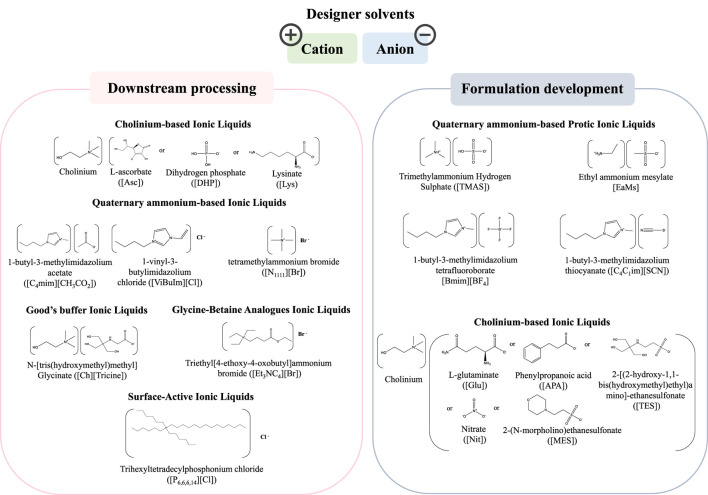
Chemical structures and nomenclature of representative ILs used in the biopharmaceutical field, namely in the downstream processing and formulation.

The outstanding properties of ILs in the biomedicine field have spurred a high interest within the scientific community (Kianfar and Mafi, 2020; Curreri et al., 2021; Bernardo et al., 2022). ILs are organic salts displaying a set of relevant properties, e.g., tailored polarity, non-flammability and non-volatility at ambient conditions, low-vapor or negligible pressure, high thermal and chemical stabilities and high solvation ability, making them attractive for a variety of applications (Castro et al., 2019; Kianfar and Mafi, 2020; Jadhav et al., 2021). ILs are considered “designer solvents” since their cations and anions can be carefully selected to provide a variety of compounds with unique physicochemical properties and biological activity (Castro et al., 2019; Curreri et al., 2021; Jadhav et al., 2021; Bernardo et al., 2022). In the biomedical and pharmaceuticals fields, ILs were mostly investigated to develop anti-cancer drugs (Yen and Chu, 2004), antimicrobial compounds (Pernak et al., 2003), and controlled drug release systems (Jaitely et al., 2008), to improve the catalytic efficiency of enzymes and to increase the thermal stability of proteins (Erbeldinger et al., 2000; Baker et al., 2004; Kumar et al., 2017; Schindl et al., 2019), and to act as excipients in synthetic drugs formulation to improve the solubility, permeability, activity, bioavailability, and stability (Moniruzzaman et al., 2010; Pedro et al., 2020; Lin et al., 2021). The application of ILs in the pharmaceutical field regarding their use with small molecules and synthetic drugs has been comprehensively reviewed elsewhere (Pedro et al., 2020) and is out of the scope of the current review. Here, we will focus on the application of ILs in the production of biopharmaceuticals, a field that is still in its infancy, but evolving at a high pace (Lin et al., 2021). The chemical structures and nomenclature of representative ILs used in the downstream processing and formulation of biopharmaceuticals, and addressed in the works herein reviewed, are compilated in Figure 4.

### Ionic liquids in the downstream processing of biopharmaceuticals

Several IL-based approaches, namely liquid-liquid and solid-liquid extractions, have been proposed for the efficient recovery and purification of a myriad of compounds, such as therapeutic enzymes (Santos et al., 2018), antibodies (Taha et al., 2015; Ferreira et al., 2016; Mondal et al., 2016; Ramalho et al., 2018; Capela et al., 2019; Vicente et al., 2022), nucleic acids (Pereira et al., 2021), viruses (Marchel et al., 2019), and interferons (Castro et al., 2020). Liquid-liquid extraction based on ILs has been proposed for the recovery and purification of a variety of biopharmaceuticals, most of them relying on the use of IL-based Aqueous Biphasic Systems (IL-ABS). In ABS the IL can either act as a phase-forming component (Taha et al., 2015; Mondal et al., 2016; Ramalho et al., 2018; Song et al., 2018; Xu et al., 2018; Capela et al., 2019; Magri et al., 2019; Quental et al., 2019; Vicente et al., 2019; Vicente et al., 2022) or as an adjuvant (Ferreira et al., 2016; Santos et al., 2018; Marchel et al., 2019; Castro et al., 2020). In addition to IL-ABS, other liquid-liquid strategies have been reported, namely Thermoresponsive Aqueous Micellar Two-Phase Systems (AMTPS) and Three-Phase Partitioning (TPP) systems. Representative works regarding the recovery and purification of biopharmaceuticals using liquid-liquid approaches based on ILs are summarized in Table 1. ILs abbreviations are provided in the footnote of Table 1.

**TABLE 1:** Representative works regarding the recovery and purification of biopharmaceuticals using liquid-liquid approaches based on ILs.

Biopharmaceutical	Biological source	Recovery/Purification system	IL	Role of IL	System composition	Recovery yield and purity	References
Immunoglobulin G (IgG) antibody	Rabbit serum	Aqueous biphasic systems (ABS)	Cholinium-based ILs, comprising [Ch][Gly], [Ch][Pyr], [Ch][Asc], [Ch][D-Gal] and [Ch][Qui]	Phase-forming component	30 wt% PPG 400 + 25 wt% IL+ 45 wt% IgG	Rabbit serum/pure IgG: 85%/100% recovery yield +∼58% purity enhancement	Mondal et al. (2016)
IgG	Rabbit serum	ABS	Cholinium-based ILs, comprising [Ch][Lac], [Ch][Gly], [Ch][Prop], [Ch][But], [Ch]Cl, [Ch][Bit], [Ch][DHCit], [Ch][DHP] and [Ch][Ac]	Phase-forming component	45 wt% PPG 400, 30 wt% PBS +25 wt% IL	Rabbit serum IgG: >80% recovery yield/49% purity	Ramalho et al. (2018)
IgG	Rabbit serum	ABS	Imidazolium, ammonium and phosphonium-based ILs comprising [C_4_mim][N(CN)_2_], [C_4_mim][CH_3_CO_2_], [C_4_mim]Cl, [C_4_mim]Br [N_1111_]Br and [N_4444_]Br	Adjuvant	25 wt% PEG 400 + 25 wt% C_6_H_5_K_3_O_7_/C_6_H_8_O_7_buffer +5 wt% IL + 45 wt% rabbit serum	42–47% recovery yield/21–26% purity	Ferreira et al. (2016)
Avian immunoglobulin Y (IgY) antibody	Water-Soluble Protein Fraction (WSPF) from chicken egg yolk	ABS	Good’s buffer ILs (GB-ILs) comprising [Ch][MES], [Ch][TES], [Ch][Tricine], [Ch][CHES] and [Ch][HEPES]	Phase-forming component	50 wt% PPG 400 +7–10 wt% IL+ 40–43 wt% WSPF	-	Taha et al. (2015)
Anti-Interleukin-8 (Anti-IL-8) monoclonal antibodies (mAbs)	Chinese Hamster Ovary (CHO) cell culture supernatants	ABS, Three-Phase Partitioning (TPP) systems and hybrid processes combining ultrafiltration	Glycine-Betaine Analogues ILs (AGB-ILs), comprising [Et_3_NC_4_]Br, [Pr_3_NC_4_]Br, [Bu_3_NC_4_]Br and [MepyrNC_4_]Br	Phase-forming component	15 wt% K_2_HPO_4_/KH_2_PO_4_+ 25,30, and 40 wt% IL+ 37.5 wt% CHO cell culture supernatant	ILs-ABS: 100% recovery yield/1.6 purification factor	Capela et al. (2019)
						ILs-TPP: 41% recovery yield/2.7 purification factor (60.9% purity)	
Recombinant interferon alpha-2b (IFNα-2b)	*E. coli* lysates	ABS	[C_2_mim]Cl, [C_4_mim]Cl, [C_6_mim]Cl, [C_4_mim]Br, [C_4_mim][CH_3_COO], [N_4444_]Cl and [Ch]Cl	Adjuvant	15 wt% phosphate salt +30 wt% PEG600 + 30 wt% PPG400 + 5 wt% IL	6.77 purification factor	Castro et al. (2020)
L-asparaginase	*E. coli* BL21 (DE3)	ABS	Imidazolium-based ILs, comprising [C_4_mim]Cl, [C_4_mim][DMP], [C_4_mim][CH_3_SO_3_] and [C_4_mim][CF_3_SO_3_]	Adjuvant	15 wt% PEG 6000 + 15 wt% citrate buffer +5 wt% IL	87.9% recovery yield/20.09 purification factor	Santos et al. (2018)
L-asparaginase	Aqueous solution of L-Asparaginase	ABS	[Ch]Cl and [Ch][Ac]	Phase-forming component	50 wt% PEG 600 + 30 wt% IL, 30 wt% PPG 400 + 16 wt% IL +	—	Magri et al. (2019)
Green fluorescent protein	BL21 (DE3) pLysS	ABS	DIMCARB	Phase-forming component	42 wt% PPG 1000 + 4.4 wt% DIMCARB +10% wt feedstock pH 8	98.8% recovery yield	Song et al. (2018)
enveloped Hepatitis C virus pseudoparticles (HCVpp)	HEK 293 (ATCC CRL-1573) cell supernatants	ABS	Imidazolium and bromide-based ILs, comprising [C_4_mim]Cl, [C_4_mim][CH_3_O_2_] and [N_4444_]Br	Adjuvant	PEG 400 + citrate buffer +5 wt% IL	40–70% purity	Marchel et al. (2019)
Ribonucleic acid (RNA)	Bacterial lysate	ABS	Amino-acid-based ILs (AA-ILs), comprising [Ch][Lys], [Ch][Arg], [Ch][Glu] and [Ch][Asp]	Phase-forming component	20 wt% PPG 400 + 20 wt% IL + 60 wt% RNA	—	Quental et al. (2019)
Salmon DNA	Standard aqueous solution containing cytochrome c	ABS	Betaine-based ILs, [Be][For], [Be][Ac], [Be][Prop], [Be][Buty]	Phase-forming component	[TBAB][PPG 400] + [Be][For] + 2 mg DNA +2 mg Cyt c	—	Xu et al. (2018)
IgG and Human Serum Albumin (HSA)	Human expired plasma	AMTPS	Surface-Active ILs (SAILs), comprising [C_14_mim]Cl and [P_4,4,4,14_]Cl	Co-surfactant	15.2 wt% Tergitol 15-S-7 + 0.3 wt% SAIL +10 wt% plasma	1.14-fold purification of IgG and 1.36-fold purification for HSA	Vicente et al. (2019)
IgY	Water-Soluble Protein Fraction (WSPF) from chicken egg yolk	Thermoresponsive AMTPS	Surface-Active ILs (SAILs), comprising [C_10_mim]Cl, [C_12_mim]Cl,[C_14_mim] Cl, [C_16_mim]Cl, [C_18_mim]Cl, [P_6,6,6,14_]Cl, [P_6,6,6,14_]Br, [P_6,6,6,14_][Dec] and [P_6,6,6,14_][TMPP]	Co-surfactant	20 wt% Triton X-114 + 0.3 or 0.5 wt% SAIL +25 wt% WSPF + McIlvaine buffer pH 6.0	69% purity	Vicente et al. (2022)
Hexahistidine-tagged (His-tagged) proteins	*E. coli.* crude BL21 (DE3)	Adsorption	Triazacyclononane-based IL namely tacn-attached 6,7-dihydro- 5H-pyrrolo [1,2-a]imidazolium and [Bmim][NTf_2_]	Chelating ligand	—	—	Ren et al. (2015)

Abbreviations: [Be][For], betaine formate; [Be][Ac], betaine acetate; [Be][Prop], betaine propionate; [Be][Buty], betaine butyrate; [C_2_mim][C_2_SO_4_], 1-ethyl-3-methylimidazolium ethylsulfate; [C_2_mim][(C_2_H_5_)_2_PO_4_], 1-ethyl-3-methylimidazolium diethyl phosphate; [C_2_mim][HSO_4_], 1-ethyl-3-methylimidazolium hydrogen sulphate; [C_6_mim]Cl, 1-hexyl-3-methylimidazolium chloride; [P_4,4,4,14_]Cl, tributyltetradecylphosphonium chloride; [C_10_mim]Cl, 1-decyl-3-methylimidazolium chloride; [C_12_mim][Cl], 1-dodecyl-3-methylimidazolium chloride; [C_14_mim]Cl, 1-tetradecyl-3-methylimidazolium chloride; [C_16_mim]Cl, 1-hexadecyl-3-methylimidazolium chloride; [C_18_mim]Cl, 1-octadecyl-3-methylimidazolium chloride; [P_6,6,6,14_]Cl, trihexyltetradecylphosphonium chloride; [P_6,6,6,14_]Br, trihexyltetradecylphosphonium bromide; [P_6,6,6,14_][Dec], trihexyltetradecylphosphonium decanoate; [P_6,6,6,14_][TMPP], trihexyltetradecylphosphonium bis (2,4,4-trimethyl (pentyl)phosphinate; [C_4_mim][N(CN)_2_], 1-butyl-3-methylimidazolium dicyanamide; [C_4_mim][CH_3_CO_2_], 1-butyl-3-methylimidazolium acetate; [C_4_mim]Cl, 1-butyl-3-methylimidazolium chloride; [C_4_mim]Br, 1-ethyl-3-methylimidazolium bromide; [N_1111_]Br, tetramethylammonium bromide; [N_4444_]Br, tetrabutylammonium bromide; [Ch][Gly], cholinium glycolate; [Ch][Pyr], cholinium pyruvate; [Ch][Asc], cholinium-L-ascorbate; [Ch][D-Gal], cholinium-D-galactouronate; [Ch][Qui], cholinium-D-(-)-quinate; [Ch][Lac], cholinium lactate; [Ch][Prop], cholinium propanoate; [Ch][But], cholinium butanoate; [Ch][Abt], cholinium abietate; [Ch][C_3_C], cholinium coumarine-3-carboxylate; [Ch][Gen], cholinium gentisate; [Ch][MES], cholinium 2-(N-morpholino)ethanesulfonate; [Ch][TES], cholinium 2-[(2-hydroxy-1,1-[bis(hydroxy methyl)ethyl)amino]ethane sulfonate; [Ch][Tricine] , cholinium N-[tris(hydroxymethyl)methyl]glycinate; [Ch][CHES], cholinium 2-cyclohexylamino)ethanesulfonate; [Ch][HEPES], cholinium 2-[4-(2-hydroxyethyl)piperazin-1-yl]ethanesulfonate; [Ch]Cl, cholinium chloride; [Ch][Bit], cholinium bitartrate; [Ch][DHCit], cholinium dihydrogen citrate; [Ch][DHP], cholinium dihydrogen phosphate; [Ch][Ac], cholinium acetate; DIMCARB, *N,N*-dimethylammonium *N’,N’*-dimethylcarbamate; [C_4_mim][CH_3_O_2_], 1-butyl-3-methylimidazolium acetate; [Ch][Lys], cholinium L-lysinate; [Ch][Arg], cholinium L-argininate; [Ch][Glu], cholinium L-glutaminate; [Ch][Asp], cholinium DL-aspartate; [TBAB], tetrabutylammonium bromide; [Et_3_NC_4_]Br, triethyl[4-ethoxy-4 oxobutyl]ammonium bromide; [Pr_3_NC_4_]Br, tri(n-propyl)[4-ethoxy-4-oxobutyl]ammonium bromide; [Bu_3_NC_4_]Br, tri(n-butyl)[4- ethoxy-4- oxobutyl]ammonium bromide; [MepyrNC_4_]Br, N-(1- methylpyrrolidyl-4-ethoxy-4-oxobutyl)ammonium bromide; [C_2_mim]Cl, 1-Ethyl-3-methylimidazolium chloride; [C_4_mim]Cl, 1-Butyl-3-methylimidazolium chloride; [C_6_mim]Cl, 1-Hexyl-3-methylimidazolium chloride; [C_4_mim]Br, 1-Butyl-3-methylimidazolium bromide; [C_4_mim][CH_3_COO], 1-butyl-3-methylimidazolium acetate; [C_4_mim][CH_3_SO_3_], 1-butyl-3-methylimidazolium methanesulfonate; [C_4_mim][CF_3_SO_3_], 1-butyl-3-methylimidazolium triflate, and [Bmim][NTf_2_], 1-butyl-3-methylimidazolium bis(trifluoromethylsulfonyl)imide.

**FIGURE 5:**
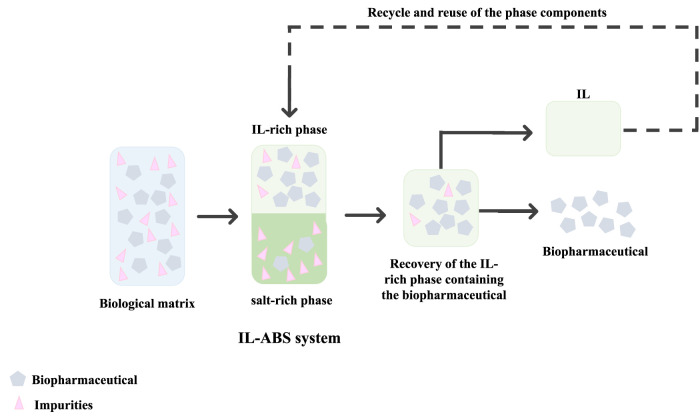
Overview of a representative liquid-liquid extraction process using IL-ABS for the recovery and purification of biopharmaceuticals.

ABS comprise two non-miscible aqueous-rich phases, formed by polymer-polymer, polymer-salt, or salt-salt combinations (Carapito, 2019; Castro et al., 2019; Bento et al., 2021). Above particular concentrations of the phase-forming components, the systems separate into two coexisting phases, each of them enriched in one component (Carapito, 2019; Castro et al., 2020). ABS display high performance, high biocompatibility, low energy consumption, allow continuous operation, and are of low-cost. By including ILs as phase-forming component/adjuvant, ABS may be designed to display different polarities and affinities to target compounds (Carapito, 2019). In addition, being aqueous systems, ABS maintain the stability of biological compounds during downstream processing. A schematic representation of a liquid-liquid extraction process using IL-ABS for the recovery and purification of biopharmaceuticals is given in Figure 5.

Mondal et al. (2016) investigated polymer-salt-based ABS formed by a combination of polypropylene glycol (PPG 400), phosphate-buffered saline (PBS) and several biobased cholinium-containing ILs, for the recovery and purification of immunoglobulin G (IgG) from rabbit serum. Systems comprising [Ch][Pyr], [Ch][Gly], [Ch][D-Gal], [Ch][Qui], and [Ch][Asc] ILs were able to form ABS and to completely extract IgG to the IL-rich phase in one-step. The [Ch][Asc]-based ABS was the most promising system, allowing a 58% enhancement in the IgG purity compared with its purity in rabbit serum samples. This study pioneeringly demonstrated the potential of ILs as phase-forming components of ABS to extract and purify antibodies. Similarly, Ramalho et al. (2018) prepared ABS formed by PPG 400 and several cholinium-based ILs, which allowed to extract commercial IgG to the IL-rich phase with extraction efficiencies ranging from 93 to 100% and recovery yields ranging from 20 to 100%. ABS formed by [Ch][Lac] and [Ch][DHP] ILs presented extraction efficiencies of 100% and allowed to purify IgG to the IL-rich phase, in one step, with 47 and 49% purity, respectively. A subsequent ultrafiltration step further increased the IgG purity up to 66% (Ramalho et al., 2018) - the highest value obtained so far for this type of system. Ferreira et al. (2016) used polymer-salt-based ABS composed of polyethylene glycol 400 g mol^−1^ (PEG 400), a buffered salt and several imidazolium-, quaternary ammonium-, and phosphonium-based ILs acting as adjuvants, for the extraction and purification of IgG from rabbit serum. ABS without ILs were able to recover IgG with 19% purity and 42% of recovery yield. The IL addition to the ABS allowed 100% extraction of IgG to the polymer-rich phase in one step, being the [C_4_mim][CH_3_CO_2_]-based ABS the best system with a recovery yield of 46% and a purity enhancement of 37% when compared to the ABS without IL. This study showed that low amounts of ILs (5 wt%) can be used in the preparation of ABS for antibody purification.

Immunoglobulin Y (IgY) antibodies, found in chicken’s egg yolk, are an important class of antibodies for therapeutic applications; however, since they derive from a complex matrix, a sequence of several steps for their purification is generally required, and mostly resorting to chromatography (Vicente et al., 2022). Taha et al. (2015) prepared polymer-salt-based ABS formed by PPG 400 and several Good’s buffer ILs (GB-ILs) to recover and purify IgY from the Water-Soluble Protein Fraction (WSPF) of egg yolk (Taha et al., 2015). It was shown that GB-ILs present a high ability to form ABS with PPG 400, in which IgY extraction efficiencies between 79 and 94% were obtained (Taha et al., 2015). Furthermore, ABS containing [Ch][Tricine] and [Ch][HEPES] GB-ILs led to the highest IgY extraction efficiencies with values above 90% achieved in a single step (Taha et al., 2015). Herein, the self-buffering nature of GB-ILs allowed to keep the pH within the physiological range without the addition of external buffers, contributing to maintain the stability of biopharmaceuticals.

The previously described studies investigated the use of ABS to purify polyclonal antibodies. However, mAbs represent the largest fraction of the biopharmaceuticals market. They are currently used in immunization, vaccination, and treatment of life-threatening diseases; nevertheless, their recovery and purification from their complex biological sources, i.e., cell culture media, also require a multi-step and expensive approach (Capela et al., 2019). To surpass these limitations and decrease all the associated burdens, Capela et al. (2019) used ABS based on glycine-betaine analogue ILs (AGB-ILs) to isolate anti-interleukin-8 (anti-IL-8) mAbs from Chinese Hamster Ovary (CHO) cell culture supernatants. Together with ABS, other strategies were also assessed, *i.e.*, TPP systems and hybrid strategies of ABS and TPP systems with ultrafiltration. ABS containing AGB-ILs allowed the recovery of the target biomolecule to the IL-rich phase with an overall yield of 100% and a purification factor of more than 1.6 (Capela et al., 2019). When the IL-based TPP system was applied, anti-IL-8 mAbs were recovered in the ABS interface with a lower yield (41%) but with an increased purification factor of 2.7 (60.9% purity) (Capela et al., 2019). Competitive enzyme-linked immunosorbent assays (ELISAs) were performed for the IL-rich phase and the precipitate after the last ultrafiltration step, as well as for the CHO cell culture for comparison purposes. It was shown that mAbs were able to maintain their biological activity after all purification and recovery stages. The recyclability of ILs was finally evaluated, showing that the separation performance was not significantly changed during three separation cycles.

Interferon alpha-2b (IFNα-2b) is an important therapeutic product in the treatment of chronic Hepatitis C and hairy cell leukaemia; however, its downstream processing is hindered by the high cost of conventional purification strategies (Castro et al., 2020). Castro et al. (2020) used ABS composed of two polymers, namely PEG of 600 g mol^−1^ (PEG 600) and PPG 400, and several ILs acting as adjuvants, for the purification of IFNα-2b from *E. coli* lysates. The addition of ILs to the ABS enhanced the IFNα-2b purification factor from 2.28 to 6.77, in which the biomolecule preferentially migrated to the PEG-rich phase (also the phase enriched in the IL) (Castro et al., 2020). Furthermore, ILs containing aromatic cations combined with high hydrogen basicity anions boosted the purity level of the protein, while maintaining its secondary structure. The IFNα-2b recovered in the PEG-rich phase was shown to be immunologically active.

L-asparaginase, also known as L-asparagine amidohydrolase enzyme, is a biopharmaceutical mostly produced by *E. coli* with therapeutic activity against acute lymphoblastic leukaemia (ALL) in humans and canine lymphosarcoma (Santos et al., 2018). Aiming to address the purification of L-asparaginase from crude enzyme extract, Santos et al. (2018) used ABS composed of PEG, citrate buffer, and several imidazolium-based ILs acting as adjuvants, combined with cell membrane permeabilization. The ABS comprising the [C_4_mim][CH_3_SO_3_] IL was identified as the best system for the enzyme recovery to the PEG-rich phase, in which a yield of approximately 87.9% and a purity factor of 20.09 were obtained (Santos et al., 2018). Moreover, the best results were achieved when ABS comprising ILs were combined with a previous enzyme purification step through ammonium sulphate precipitation, allowing to obtain a higher purification factor (Santos et al., 2018). In a different study, Magri et al. (2019) studied the partition of L-asparaginase using a series of polymer/salt-based ABS including, among others, PEGs of different molecular weights (PEG 600, polyethylene glycol of 2,000 g mol^−1^ (PEG 2000)) and PPG 400, to understand the mechanisms behind the separation of the enzyme. The following ABS were studied: PEG 600 + phosphate buffer, PEG 2000 + phosphate buffer, PEG 600 + citrate buffer, PEG 600 + sodium sulphate, PEG 600 + [Ch][Cl], PEG 2000 + citrate buffer, PEG 2000 + sodium sulphate, PPG 400 + [Ch][Cl], and PPG 400 + [Ch][Ac]. The obtained results demonstrated that by changing the relative hydrophobicity of the polymers, the systems induce the inversion of the enzyme partitioning, being preferentially partitioned to the polymer-rich phase in ABS composed of IL-PEG 600 since it is constituted by the polymer that is more hydrophilic, and to the IL-rich phase using PPG 400-based ABS, formed by the polymer that is more hydrophobic. PEG 600-IL-based ABS lead to a decrease in its biocatalytic activity, whereas systems comprising PPG 400 maintained the relative activity of L-asparaginase around 100%. Then, L-asparaginase purification from the fermentation broth was conducted and the purification performance of the systems in study was optimized. It was found that the ABS comprising PEG 2000 and phosphate buffer was the most effective with a purification factor of *ca.* 2.4, while preserving the activity of L-asparaginase.

Green fluorescent protein (GFP) is a protein exhibiting bright green fluorescence when exposed to light in the blue to ultraviolet range. Therefore, it can be used as a powerful biomarker and visualization tool in diverse cellular processes. Song et al. (2018) investigated the recovery of GFP from *E. coli* using ABS constituted by PPG 1000 and the *N,N*-dimethylammonium *N′,N′*-dimethylcarbamate (DIMCARB) IL. This IL may represent a more environmentally friendly option, since it presents a simpler and cheaper synthesis route, as well as enhanced biodegradability, and biocompatibility. Furthermore, this CO_2_-based alkyl carbamate IL can be distilled at a relatively low temperature under vacuum, allowing a simple recovery of the IL for subsequent extraction processes. The authors found that GFP was preferentially partitioned to the IL-rich phase in ABS composed of 42 wt% of PPG 1000 and 4.4 wt% of DIMCARB, with a GFP selectivity of 1.2 and 98.8% of yield. The system could be scaled up 50 times without compromising the purification performance, and DIMCARB was successfully recycled and reused in three consecutive GFP purification processes. The process did not affect the GFP stability. Also, Soares et al. (2021) investigated the continuous purification of the variant enhanced GFP (EGFP) through a fast centrifugal partition chromatography (FCPC) using ABS formed by polyethylene glycol (PEG 8000), sodium polyacrylate (NaPA 8000), and sodium sulphate (Na_2_SO_4_) acting as an electrolyte. The most effective ABS, *i.e.*, the one formed by 15 wt% PEG 8000 with 4.5 wt% NaPA 8,000 and 2.5 wt% Na_2_SO_4_ was selected and applied for FCPC. This combination of FCPC with ABS led to an EGFP recovery yield of 82.3% and a purification of 89.93%.

Virus-like particles (VLPs) are also considered promising biopharmaceuticals, particularly in VLP-based vaccination and cancer therapy; however, conventional VLPs separation techniques (gradient ultracentrifugation, ultrafiltration, precipitation, size-exclusion chromatography) are still challenged by the low yield, low selectivity, and difficulties in scale-up, limiting their widespread application (Marchel et al., 2019). To overcome these drawbacks, Marchel et al. (2019) performed a high-throughput screening on a liquid handling station to select the most appropriate ILs to be used as adjuvants in polymer-salt-based ABS, followed by the application of the prepared systems for the extraction and purification of enveloped Hepatitis C virus pseudoparticles (HCVpp), a VLP able to infect liver cells resulting in liver disease and cirrhosis, and ultimately in hepatic failure and death (Marchel et al., 2019). ABS composed of PEG 400, citrate buffer, and bromide-based ILs acting as adjuvants, allowed 100% extraction of the target VLPs to the PEG-rich phase (Marchel et al., 2019). Moreover, when no IL was present in the ABS, a purity degree of 53% was obtained, contrasting with the purity range of 40–70% obtained when ILs were used as adjuvants (Marchel et al., 2019). This study demonstrated that it is possible to increase the purity of viral particles by using appropriate ILs as adjuvants able to modulate the properties of the ABS, thus requiring lower amounts of ILs.

Nucleic acids, including RNAs, play key roles in the diagnosis and treatment of diseases. Current methods available for their isolation include phenol/chloroform-based extraction and Solid-Phase Extraction (SPE), whereas for their purification chromatographic techniques (e.g., gel filtration, reverse-phase, ion-pairing, and ion-exchange chromatography) and denaturing polyacrylamide gel electrophoresis are usually applied (Ferreira et al., 2000; Quental et al., 2019; Neves et al., 2020; Pereira et al., 2021). In addition to resorting to hazardous organic solvents, these conventional strategies are time-consuming, laborious, and lead to low recovery yields (Neves et al., 2020). With this challenge in mind, Quental et al. (2019) used ABS formed by PPG- 400 and several cholinium-based ILs comprising anions derived from amino acids (AA-ILs) to purify ribonucleic acid (RNA) from bacterial lysate samples, while preserving its stability. These ILs were selected given the previously shown potential of columns modified with amino acids to purify nucleic acids (Pereira et al., 2014a; Pereira et al., 2014b). RNA was successfully extracted from bacterial lysate samples to the IL-rich phase without compromising its integrity and stability. Moreover, it was possible to recover RNA from the IL-rich phase through ethanol precipitation, as well as to recover and reuse the phase-forming components of the ABS in new purification steps. Xu et al. (2018) investigated ABS comprising deep eutectic solvents (DES) and betaine-based ILs to extract DNA from salmon testes. The DES used in ABS preparation were formed by different molar ratios of hydrogen bond acceptor (HBA), hydrogen bond donor (HBD) and water (HBA:HBD:H_2_O). The ability of these systems to extract DNA was amplified by [TBAB][PPG 400] and by the IL [Be][For], being observed that experimental parameters such as temperature, mass of IL, mass of DES, extraction time, pH, and ionic strength have a significant effect on the DNA extraction efficiency. Under these conditions, proteins with higher isoelectric points (pI) such as cytochrome c (pI = 10) are preferentially partitioned to the DES-rich phase, thus allowing their separation from DNA, which is preferentially enriched in the IL-rich phase. Furthermore, it was shown that the structural integrity and chemical stability of DNA, before and after its extraction, were preserved.

As mentioned previously, in addition to ABS, other liquid-liquid systems such as AMTPS have also been investigated for purification purposes. AMTPS are mainly constituted by water and a surfactant, resulting in two-phase liquid systems at given temperatures and surfactant concentrations (Vicente et al., 2022). If properly designed, AMTPS exhibit higher percentages of water in comparison to traditional ABS, being therefore able to preserve the original conformation and biological activity of biomolecules. Vicente et al. (2019) investigated the potential of using AMTPS formed by non-ionic surfactants, namely Triton X-114 or Tergitol 15-S-7, to concurrently separate IgG and Human Serum Albumin (HSA) from human expired plasma. After optimization of the conditions with the previous AMTPS systems, the ability of mixed AMTPS composed of Tergitol 15-S-7 and several surface-active ionic liquids (SAILs) acting as cosurfactants to tailor the IgG and HSA partition between the two phases was investigated. In this way, the mixed AMTPS formed by Tergitol 15-S-7 and tributyltetradecylphosphonium chloride IL as the cosurfactant at pH 8.0 was able to enhance the simultaneous separation of IgG and HSA to the opposing phases of the system. A 1.14-fold purification of IgG and a 1.36-fold purification for HSA were obtained in the surfactant-poor and the surfactant-rich phases, respectively. Vicente et al. (2022) studied the potential of AMTPS formed by the non-ionic surfactant Triton X-114 and several imidazolium- and phosphonium-based SAILs acting as co-surfactants for the isolation and purification of IgY antibodies from the WSPF of chicken’s egg yolk. In this study, the AMTPS comprising the [C_18_mim]Cl SAIL was able to enhance the recovery of IgY to the surfactant-poor phase, in a single-step, with a purity of 69%, whereas traditional AMTPS allowed a purity of 54% (Vicente et al., 2022). Moreover, it was shown that after applying three successive cycles of purification using consecutively the surfactant-poor phase of the [C_14_mim]Cl-based AMTPS, the purity improved to up to 73% (Vicente et al., 2022).

**FIGURE 6:**
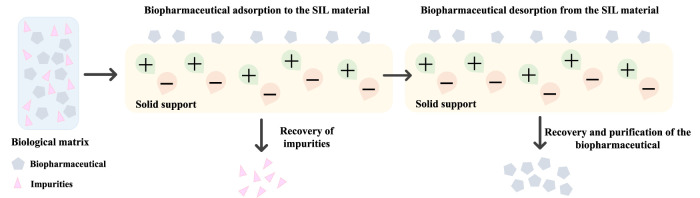
Overview of a representative SPE process using SILs for the purification of biopharmaceuticals.

Although the majority of works regarding the recovery and purification of biopharmaceuticals by IL-based approaches are established on liquid–liquid extraction, pertinent scientific evidence has been recently published showing the potential of solid-phase extraction (SPE) to enhance the downstream processing of therapeutic biomolecules. The field of SPE involving IL-modified materials, known as Supported Ionic Liquids (SILs), is gaining increasing traction. SILs are usually obtained by the immobilization of ILs in a suitable solid support, e.g., silica or polymeric support, combining the ILs properties with the advantages exhibited by materials (e.g., high specific surface area, and mechanical properties, among others) (Lemus et al., 2011; Pedro et al., 2019; Bento et al., 2021). This led to a modified material with adjustable properties that are coupled to an efficient immobilization on a confined environment. They can be considered “designer surfaces” since the properties of ILs are transferred to the solid surfaces, thus constituting attractive materials (Fehrmann et al., 2014). Depending on the interactions established between the IL and the support material, the immobilization of these solvents can be attained by two pathways (Tavares et al., 2019): physical confinement of the IL in the materials, i.e., physisorption, through van der Waals and dipole forces; or by 2) covalent bonding between the support and the IL, i.e., chemisorption (Tavares et al., 2019; Bento et al., 2021). SILs obtained by the first approach are generally present as a multilayer in which properties of the neat ILs are almost maintained, being mainly applied in gas capture (Giacalone and Gruttadauria, 2016; Bento et al., 2021). In contrast, by applying a chemisorption process to immobilize the IL onto the support through covalent binding, the ILs leaching from the material will be avoided (Pedro et al., 2019), being thus relevant to be applied in SPE dealing with liquid samples. Figure 6 depicts the general application of SILs in the SPE of biopharmaceuticals. SILs have been applied in SPE and chromatography to separate proteins and enzymes by taking advantage of some specific properties of ILs, in which high adsorption capacities and selectivity were achieved (Itoh and Koo, 2019). Most of these studies involve SILs in which silica or polymers were used as supports; however, it is important to highlight that this field is still in the early stages of development, with most works published dealing with the adsorption of model proteins from aqueous solutions (Bento et al., 2021). Few scientific works reported the application of SILs in the separation or recovery of biomolecules from complex biological mixtures, including nucleic acids (Neves et al., 2020; Pereira et al., 2021), bovine serum albumin (Liu et al., 2014; Song et al., 2016; Jia et al., 2019), haemoglobin (Zhao et al., 2013), cytochrome-c (Yuan et al., 2012; Liu et al., 2014), lysozyme (Yuan et al., 2012; Liu et al., 2014), and bovine haemoglobin (Liu et al., 2014; Song et al., 2016). Representative works regarding the recovery and purification of biopharmaceuticals using solid-liquid approaches based on SILs are compilated in Table 2. ILs abbreviations are provided in the footnote of Table 2.

**TABLE 2:** Representative works regarding the recovery and purification of biopharmaceuticals using solid-liquid approaches based on SILs.

Biopharmaceutical	Source	Solid support	IL	References
Ribonucleic acid (RNA)	*E. coli DH5α*	Silica-modified materials	Chloride-based ILs comprising [C_3_C_1_im]Cl, [N_3222_]Cl, [N_3114_]Cl, and [N_3888_]Cl	Pereira et al. (2021)
Small RNAs, ribosomal RNA, and genomic DNA	*E. coli DH5*α *lysate*	Commercial macroporous resin named Toyopearl AF-Epoxy-650M	[C_3_C_1_im]Cl	Neves et al. (2020)
Immunoglobulin G (IgG)	Human serum, rabbit serum and Chinese hamster ovary (CHO) cell culture supernatants	Silica-modified materials	[C_3_C_1_im]Cl, [N_3444_]Cl and [N_3888_]Cl	Capela et al. (2023)
Lysozyme, cytochrome-c, and haemoglobin	Standard aqueous solutions	Polyvinyl chloride (PVC) materials	Imidazolium-based ILs comprising [Nmim]Cl	Shu et al. (2010)
Lysozyme, cytochrome-c, bovine serum albumin, bovine haemoglobin and equine myoglobin	Standard aqueous solutions	Macroporous polymer material modified	Imidazolium-based ILs comprising [ViBuIm]Cl	Yuan et al. (2012)
Lysozyme, bovine serum albumin, trypsin and ovalbumin	Standard aqueous solutions	Magnetic multiwall CNTs	Dual hydroxyl functional ILs	Chen et al. (2015)
Lysozyme	Standard aqueous solutions	Graphene oxide	Guanidinium-based IL comprising [diBOHTMG]Cl	Ding et al. (2015)

Abbreviations: [C_3_C_1_im]Cl, 1-methyl-3-propylimidazolium chloride; [N_3222_]Cl, triethylpropylammonium chloride; [N_3444_]Cl, propyltributylammonium chloride; [N_3114_]Cl, dimethylbutylpropylammonium chloride; [N_3888_]Cl, trioctylpropylammonium chloride; [Nmim]Cl, N-methylimidazole; [ViBuIm]Cl, 1-vinyl-3-butylimidazolium chloride, and [diBOHTMG]Cl, hexabutylguanidinium chloride.

Pereira et al. (2021) used silica modified with chloride-based ILs to isolate bacterial RNAs from *E. coli* lysates. SILs comprising [C_3_C_1_im]Cl and [N_3114_]Cl were the best adsorbent materials for the effective and selective isolation of the target compound, with binding capacities of 16.3 and 15.6 μmol g^−1^, respectively (Pereira et al., 2021). It is important to highlight that this remarkable applicability of ILs as chromatographic ligands results from ILs diversity in terms of the chemical structures and functional groups, allowing the establishment of a variety of interactions with the target biomolecule, such as hydrophobic and electrostatic interactions, as reviewed by Bento et al. (2021) and Bernardo et al. (2022). The potential of using macroporous polymeric supports functionalized with ILs to purify nucleic acids was reported by Neves et al. (2020). [C_3_C_1_im]Cl was identified as the most promising ligand to be used in preparative liquid chromatography. The prepared support material allowed to purify, in a single-step and with high selectivity and dynamic binding capacity, different classes of nucleic acids, namely small RNAs, ribosomal RNA, and genomic DNA, from a bacterial lysate. In this work, it was pioneeringly shown that ILs behave as multimodal ligands. Moreover, these IL-based materials were regenerated using NaOH and HCl aqueous solutions and reused without compromising their separation performance.

In addition to nucleic acids, SILs have been applied in the purification of both monoclonal and polyclonal IgG antibodies. Capela et al. (2023) investigated the use of SILs, namely with the IL ligands [C_3_C_1_im]Cl, [N_3444_]Cl, and [N_3888_]Cl in silica supports to capture and purify IgG from complex biological samples, i.e., human and rabbit sera and CHO cell culture supernatants. By using the 1- methyl-3-propylimidazolium-based supported material, IgG was directly recovered from the aqueous solution (diluted human serum) with a yield of 59% and a purity degree of 84%. On the other hand, by using the propyltrioctylamonium-based supported material, IgG was adsorbed from human serum samples onto the surface of the material with a recovery yield of 76% and a purity of 100%. The same conditions were also applied to recover IgG from other biological sources, namely rabbit serum and CHO cell culture supernatants, demonstrating the versatility and reproducibility of the used SILs. This work showed that SILs are customizable materials, in which the bind-and-elute and flowthrough-like modes can be applied just by changing the IL chemical structure.

Shu et al. (2010) prepared [Nmim]Cl/polyvinyl chloride (PVC) materials by immobilizing imidazolium cations, namely [Nmim]^+^ moieties, onto a PVC support to extract lysozyme and cytochrome-c from prepared aqueous solutions. Cytochrome c is a small mitochondrial electron transport heme protein that has been used in several bioelectrochemical and therapeutic applications, for instance as a biosensor and anticancer drug (Santos et al., 2022), whereas lysozyme is a therapeutic compound used in gastrointestinal infections (Sava, 1996). These [Nmim]Cl/PVC materials were able to adsorb both proteins with high efficiency (97–98%). Also, Yuan et al. (2012) prepared a macroporous polymeric material that was modified with [ViBuIm]Cl, to selectively adsorb cytochrome-c and lysozyme, as well as three other proteins, including bovine serum albumin, bovine haemoglobin, and equine myoglobin. The synthetized SIL presented a strong binding capacity for the proteins, in particular for lysozyme, presenting a maximum capacity of 755.1 mg g^−1^.

Carbon-based materials, including carbon nanotubes (CNTs) (Chen et al., 2015) and graphene oxide (Ding et al., 2015) have also been successfully used to immobilize ILs. Chen et al. (2015) used magnetic multiwall CNTs modified with dual hydroxyl functional ILs to extract lysozyme. The maximum adsorption capacity attained by the enzyme was 94.6 mg g^−1^, with a desorption ratio and recovery of 91.6% and 97.8%, respectively (Chen et al., 2015). For comparison purposes, the extraction of standard proteins and enzymes, namely with bovine serum albumin, trypsin, and ovalbumin, was additionally studied. It was shown that the extraction efficiency decreases in the following order: lysozyme (94.6 mg g^−1^) > trypsin (73.3 mg g^−1^) > ovalbumin (39.6 mg g^−1^) > bovine serum albumin (31.4 mg g^−1^). This tendency can be explained by the PI and the size of proteins. The pIs of bovine serum albumin (BSA) and ovalbumin are between 4.7 and 4.8, while lysozyme and trypsin pIs are, respectively, 10.8 and 10.5. Therefore, and considering the pH in study (Pereira et al., 2021), bovine serum albumin and ovalbumin will be predominantly negatively charged, whereas lysozyme and trypsin will be positively charged. In these conditions, the surfaces of the material (pI = 6.3) are negatively charged. Due to the electrostatic interactions between negatively charged and positively charged nanoparticles, the amount of lysozyme and trypsin that are extracted is higher than the amount of bovine serum albumin and ovalbumin extracted. Moreover, a small sized protein, *i.e.*, lysozyme and trypsin, makes easier its extraction. The manuscript was slightly modified accordingly (Chen et al., 2015). Ding et al. (2015) used a magnetic chitosan and graphene oxide functionalized with a guanidinium IL, namely [diBOHTMG]Cl, composite to extract lysozyme and were able to obtain a value of 38.4 mg g^−1^. Moreover, the material was easily regenerated and reused three times without significant losses on the adsorption efficiency of the protein. As highlighted previously, also this set of works was carried out with prepared aqueous solutions containing a mixture of proteins, requiring additional studies with real matrices.

Despite the limited research that has been carried out so far on the use of IL-based SPE for the recovery and purification of biopharmaceuticals, the evidence gathered up to date indicates that SILs materials could be a promising approach for this purpose, in which different types of interactions could be investigated and tailored.

### Ionic liquids in the formulation of biopharmaceuticals

**FIGURE 7:**
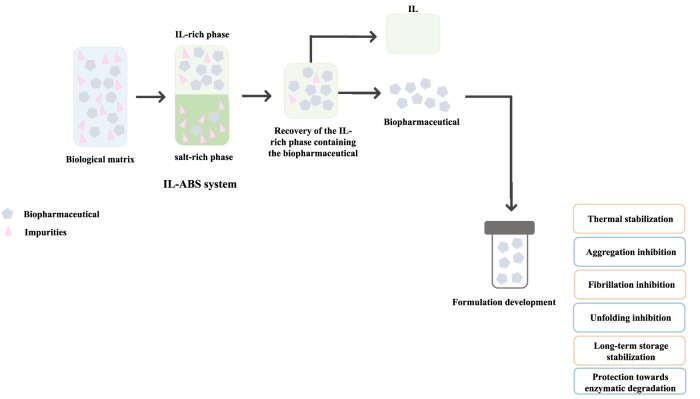
Representative integration of biopharmaceuticals’ downstream processing and formulation development stages using IL-based approaches.

IL-based approaches have been proposed to surpass the shortcomings still faced in the formulation development stage of biomanufacturing, namely to improve the stability of biopharmaceuticals, including insulin (Kumar and Venkatesu, 2013; Kumar and Venkatesu, 2014; Todinova et al., 2016; Banerjee et al., 2018; Guncheva et al., 2019), interleukins (Foureau et al., 2012; Jagannath et al., 2018), cortisol (Jagannath et al., 2018), antibodies (Mazid et al., 2015; Ferreira et al., 2016; Reslan et al., 2018; Vicente et al., 2022), nucleic acids (Vijayaraghavan et al., 2010; Mukesh et al., 2013; Pedro et al., 2018), and viruses (Byrne et al., 2012; Lin et al., 2019). Most ILs investigated enhanced or maintained the biomolecules’ thermal and chemical stabilities, inhibiting their aggregation, denaturation, and enzymatic degradation, while promoting long-term stability and prolonged shelf-life. A flowchart illustrating the integration between downstream processing and formulation development stages of biopharmaceuticals using IL-based approaches, accompanied by the general effects of ILs in biopharmaceutical’s stability is presented in Figure 7. This integrated approach was pioneeringly proposed by Quental et al. (2019) for the extraction-preservation of RNA, discussed before.

Representative works regarding the application of ILs in the formulation of biopharmaceuticals are compilated in Table 3. ILs abbreviations are provided in the footnote of Table 3. Todinova et al. (2016) studied the effect of several imidazolium-based ILs on the stability of the monomeric form of insulin under acidic pH conditions. It was shown that, among all studied ILs, the [C_4_C_1_im]Cl IL increased the transition temperature (T_m_) of insulin from approximately 75.4°C (when no IL was present) to 86.9°C at IL concentrations of 0.3 mol L^−1^, while preserving its helicoidal structure (Todinova et al., 2016). Moreover, no aggregation events were observed in the presence of the studied ILs, demonstrating the potentiality of these solvents for the maintenance and/or enhancement of insulin stability (Todinova et al., 2016). Kumar and Venkatesu (2014) also studied the stability of insulin in the presence of imidazolium-based ILs. It was demonstrated that [C_4_C_1_im]Br and [C_4_C_1_im]Cl were able to stabilize the native form of the protein, whereas the remaining ILs, comprising [SCN]^-^, [HSO_4_]^-^ and [CH_3_COO]^-^, led to insulin denaturation (Kumar and Venkatesu, 2014). In the same line, Kumar and Venkatesu (2013) reported the use of ammonium-based protic ILs (PILs) to prevent aggregation and preserve the thermal stability of insulin. Through the application of several analytical techniques, including UV-Visible Spectroscopy, Circular Dichroism (CD), Dynamic Light Scattering (DLS) and Fluorescence Spectroscopy, it was shown that the ammonium-based IL formed by [TEAP] hindered the formation of inactive forms of the protein while maintaining its thermal stability (Kumar and Venkatesu, 2013). Also, Guncheva et al. (2019) synthetized a total of six biocompatible cholinium-based ILs containing several amino acids as anions and evaluated their effects on the secondary structure of insulin (Guncheva et al., 2019). Its T_m_ increased by 9.7 and 4.5°C in the presence of [Ch][Glu] and [Ch]_2_ [Asp] ILs, respectively (Guncheva et al., 2019). The synthetized cholinium-based ILs contributed to increasing the β-sheet conformations in comparison to the α-helix ones and led to molecular rearrangements within the protein. Furthermore, [Ch][Lys] and [Ch][Arg] inhibited insulin aggregation and fibrillation; however, it was observed a decrease in thermal stability (Guncheva et al., 2019). Finally, the ILs cytotoxicity on murine embryonic fibroblasts (3T3) (ATCC) was assessed, showing that all ILs, applied at the concentrations of 0.05 and 0.5 mmol L^−1^, did not affect fibroblast proliferation for 48 h; however, a significant reduction of the cell growth of more than 50% and a moderate anti-proliferative activity of more than 25% were achieved with [Ch][Arg] and [Ch][Glu], respectively, in the experiments in which the highest concentration was tested. Overall, it was concluded that cholinium-based ILs can be considered potential stabilizing agents for relevant therapeutic proteins, such as insulin; however, it is crucial to properly select the IL nature and concentration, when envisioning their application in the biopharmaceutical field. Still regarding the same target molecule, Banerjee et al. (Banerjee et al., 2018) showed the potential of choline and geranate (CAGE) ILs for oral insulin delivery, showing that these solvents can protect insulin from enzymatic degradation.

**TABLE 3:** Representative works regarding the use of ILs in biopharmaceutical formulations.

Biopharmaceutical	Biological source	IL	Role of IL	References
Insulin	—	Ammonium-based protic ILs (PILs) comprising [TMAS], [TEAS], [TMAP], [TEAP] and [TMAA]	Thermal stabilization and inhibition of the formation of inactive forms of the protein	Kumar and Venkatesu (2013)
Insulin	Porcine pancreas	Cholinium-based ILs in the presence of amino acids, comprising [Ch][Glu] and [Ch]_2_[Asp]	Inhibition of aggregation and fibrillation of the protein	Guncheva et al. (2019)
Insulin	Porcine pancreatic B-cells	Imidazolium-based ILs comprising [C_4_C_1_im][CH_3_COO], [C_4_C_1_im][Cl], [C_4_C_1_im][CF_3_COO], [C_4_C_1_im][SCN] and [C_4_C_1_im][N(CN)_2_]	Stabilization and improvement of the helical structure of the protein	Todinova et al. (2016)
Insulin	—	Cholinium and geranate (CAGE) ILs	Protection from enzymatic degradation	Banerjee et al. (2018)
NFKBIZ small interfering RNA (siRNA)	—	Cholinium-based ILs comprising [Ch][AGE], [Ch][ADA], [Ch][AVA], [Ch][APA] [Ch][ASA], [Ch][APP] and [Ch][ABA]	Preservation of the secondary structure of the protein	Mandal et al. (2020)
Insulin	—	Imidazolium-based ILs comprising Br^-^ and Cl^-^ anions	Stabilization of the native form	Kumar and Venkatesu (2014)
Recombinant human interleukin-2 (rhIL-2)	—	Cholinium-based ILs comprising [Ch][DHP]	Thermal stabilization	Foureau et al. (2012)
Interleukin-6 (IL-6) and cortisol antibodies	Human sweat	Cholinium and imidazolium-based ILs comprising [Ch][DHP] and [Bmim][BF_4_]	Stabilization	Jagannath et al. (2018)
Epidermal Growth Factor Receptor (EGFR) mAb	—	Cholinium-based buffered ILs (BILs) comprising [Ch][DHP]	Stabilization and inhibition of protein fragmentation	Mazid et al. (2015)
Herceptin ^®^ (trastuzumab) mAb	—	Cholinium-based ILs comprising [Ch][DHP]	Inhibition of the unfolding and	Reslan et al. (2018)
Immunoglobulin G (IgG)	Human serum	Cholinium-based ILs comprising [Ch][Ac], [Ch][Cl], [Ch][DHC] and [Ch][DHP]	Enhancement of the thermal and chemical stability, and preservation of the protein	Dhiman et al. (2022)
Immunoglobulin Y (IgY)	Water-Soluble Protein Fraction (WSPF) from chicken egg yolk	Surface-Active ILs (SAILs), comprising [C_14_mim]Cl	Stabilization	Vicente et al. (2022)
Deoxyribonucleic acid (DNA)	Salmon testes	Cholinium-based IL comprising [Ch][IAA]	Chemical and structural stabilization and inhibition of the protein degradation	Mukesh et al. (2013)
Deoxyribonucleic acid (DNA)	Salmon testes	Cholinium-based ILs comprising [Ch][Lac], [Ch][DHP] and [Ch][Nit]	Structure preservation and long-term storage stabilization	Vijayaraghavan et al. (2010)
Double stranded Deoxyribonucleic acid (dsDNA)	Salmon testes	Cholinium-, tetrabutylammonium-, tetrabutylphosphonium-, and imidazolium-based ILs comprising [N_4444_]Br, [P_4444_]Br, [C_2_C_1_im]Br, [Ch]Br, [Ch]Cl, [Ch][DHP], [Ch][Ac] and [Ch][Gly]	Preservation media	Dinis et al. (2020)
Recombinant small ribonucleic acid (RNA)	*E. coli DH5α* strain	Self-buffering cholinium-based Good’s buffers ILs (GB-ILs) comprising [Ch][HEPES], [Ch][MES], [Ch][Tricine] and [Ch][TES]	Preservation media	Pedro et al. (2018)
RNA	Bacterial lysates	Amino-acid-based ILs (AA-ILs) comprising [Ch][Lys], [Ch][Arg], [Ch][Glu] and [Ch][Asp]	Preserve stability	Quental et al. (2019)
Tobacco Mosaic Virus (TMV)	*Nicotiana tobacum* plants infected with TMV	Protic ILs comprising [EaMs], [DEaMs] and [TeaMs] anions	Improvement of the shelf-life, without structural losses	Byrne et al. (2012)
Inactivated Foot-and-Mouth Disease Virus (iFMDV)	—	Cholinium-based ILs comprising [Ch][Cl], [Ch][SO_4_] and [Ch][H_2_PO_4_]	Enhancement of the thermal and long-term stability	Lin et al. (2019)

Abbreviations: TMAS, trimethylammonium hydrogen sulphate; TEAS, triethylammonium hydrogen sulphate; TMAP–trimethylammonium dihydrogen phosphate; TEAP, triethylammonium dihydrogen phosphate; TMAA, trimethylammonium acetate; [C_4_C_1_im][CH_3_COO], 1-butyl-3-methylimidazolium acetate; [C_4_C_1_im][C], 1-Butyl-3-methylimidazolium chloride; [C_4_C_1_im][CF_3_COO], 1-butyl-3-methylimidazolium trifluoroacetate; [C_4_C_1_im][SCN], 1-butyl-3-methylimidazolium thiocyanate; [C_4_C_1_im][N(CN)_2_], 1-butyl-3-methylimidazolium dicyanamide; [Ch][AGE], cholinium geranate; [CH][ADA], cholinium dimethylacrylic acid; [Ch][AVA], cholinium iso-valeric acid; [Ch][APA], cholinium phenylpropanoic acid; [Ch][ASA], cholinium 4-phenolsulfonic acid; [Ch][APP], cholinium phenyl-phosphoric acid; [Ch][ABA], cholinium biphenyl-3-carboxylic acid; [Br]^-^, bromide anion; [Cl]^-^, chloride anion; [Ch][Glu], cholinium L-glutaminate; [Ch]_2_[Asp], cholinium L-asparaginate; [Ch][DHP], cholinium dihydrogen phosphate; [Bmim][BF_4_], 1-butyl-3-methylimidazolium tetrafluoroborate; [P_4444_][Br], tetrabutylphosphonium bromide; [N_4444_][Br], tetrabutylammonium bromide; [C_4_mim][Cl], 1-butyl-3-methylimidazolium chloride; [C_4_mim][Br], 1-ethyl-3-methylimidazolium bromide; [C_4_mim]N(CN)_2_], 1-butyl-3-methylimidazolium dicyanamide; [C_4_mim][CH_3_CO_2_], 1-butyl-3-methylimidazolium acetate; [C_14_mim]Cl, 1-tetradecyl-3-methylimidazolium chloride; [Ch][IAA], cholinium-indole-3-acetate; [Ch][Lac], cholinium lactate; [Ch][Nit], cholinium nitrate; [N_4444_]Br, tetrabutylammonium bromide; [P_4444_]Br, tetrabutylphosphonium bromide; [C_2_C_1_im]Br, 1-ethyl-3-methylimidazolium bromide; [Ch]Br, cholinium bromide; [Ch]Cl, cholinium chloride; [Ch][DHP], cholinium dihydrogen phosphate, [Ch][Ac], cholinium acetate; [Ch][Gly], cholinium glycolate; [Ch][HEPES], 2-[4-(2-hydroxyethyl)piperazin-1-yl]ethanesulfonate; [Ch][MES], cholinium 2-(N-morpholino)ethanesulfonate; [Ch][Tricine], cholinium N- [tris(hydroxymethyl)methyl]glycinate; [Ch][TES], cholinium 2-[(2-hydroxy-1, 1-bis(hydroxymethyl)ethyl)amino]-ethanesulfonate; [EaMs], ethyl ammonium mesylate; [DeaMs], diethylammonium mesylate; [TeaMs], triethylammonium mesylate; [Ch][Cl], cholinium chloride, [Ch][SO_4_], cholinium sulphate; [Ch][H_2_PO_4_], cholinium dihydrogen phosphate; [Ch][Arg], cholinium L-argininate; [Ch][Asp], cholinium DL-aspartate; [Ch][Lys], (2-hydroxyethyl) trimethylammonium (cholinium) L-lysinate.

Foureau et al. (2012) studied the effect of [Ch][DHP] on the functional and structural integrity of recombinant human interleukin-2 (rhIL-2), a protein that is used for the treatment of advanced melanoma. It was shown that for rhIL-2 formulations comprising 680 mmol L^−1^ IL, the IL was able to preserve the binding activity of rhIL-2 after a thermal treatment, in which temperatures of 23.3°C above the protein T_m_ were applied (Foureau et al., 2012). Cytotoxicity assays were also performed to evaluate the safety of [Ch][DHP], being observed that it presents a non-toxic profile against primary splenocytes or B16F10 cell lines (Foureau et al., 2012). The [Ch][DHP] IL increased the thermal stability of rhIL-2, while being non-toxic, confirming its potential to prepare safe and stable formulations of therapeutic proteins (Foureau et al., 2012). In the study conducted by Jagannath et al. (2018), the ability of [Ch][DHP] and [C_4_C_1_im][BF_4_] ILs to stabilize interleukin-6 (IL-6) and cortisol antibodies was evaluated. It was shown that upon the addition of [Ch][DHP] to the final formulation at concentrations higher than 60% (v/v), it was able to stabilize both proteins, avoiding aggregation (Jagannath et al., 2018).

In the antibody field, Reslan et al. (2018) studied the effect of [Ch][DHP] IL on the stability of Herceptin^®^ (trastuzumab) mAb. To achieve this purpose, several formulations containing different concentrations of the IL and the mAb were prepared, in the presence and in the absence of other excipient compounds that are typically used in conventional Herceptin^®^ formulations (Reslan et al., 2018). Overall, it was shown that [Ch][DHP] inhibited the aggregation and unfolding of the protein and that its stability could be significantly enhanced when the IL was combined with other excipients (Reslan et al., 2018). Moreover, in formulations containing 53% (w/v) of [Ch][DHP], the transition temperature and the onset temperature of aggregation (T_agg_) of 20 mg mL^−1^ of trastuzumab, increased by 5.6 and 10.4°C, respectively (Reslan et al., 2018). The effect of cholinium-based buffered ILs (BILs), comprising again [Ch][DHP], in the structural and chemical stability of the Epidermal Growth Factor Receptor (EGFR) mAb was also studied (Mazid et al., 2015). It was demonstrated that the introduction of BILs in the EGFR mAb formulations inhibits protein fragmentation, preserves its α-helix conformation, prolongs its stability and activity in the presence of proteinases, and maintains its biological activity during storage (Mazid et al., 2015). This study disclosed the potential of BILs as storage buffers for mAbs, decreasing the necessity to apply other preservation techniques, such as lyophilisation and refrigeration. Dhiman et al. (2022) investigated the use of cholinium-based ILs as stabilizers of polyclonal IgG antibodies. It was shown that these solvents do not lead to IgG aggregation or fragmentation, being [Ch][Ac] and [Ch]Cl the ILs with higher ability to improve the thermal stability of the protein. Vicente et al. (2022) studied the effect of SAILs-based AMTPS on the structural stability of IgY antibodies. This work disclosed that, even when IgY antibody is effectively recovered from its biological source with IL-based AMTPS, its stability is preserved.

In addition to protein-based pharmaceuticals, a set of works has shown the ability of ILs to improve the stability of nucleic acids. In the work conducted by Mukesh et al. (2013), two cholinium-based ILs were studied with DNA from salmon testes. DNA denaturation occurred when it was formulated with the [Ch][IBA] IL, whereas the introduction of the [Ch][IAA] IL enabled to dissolve DNA up to 3.5% (w/w) and to maintain its long-term chemical and structural stabilities (Mukesh et al., 2013). Similarly, Vijayaraghavan et al. (2010) demonstrated the ability of cholinium-based ILs to maintain the structural conformation and storage stability of salmon testes DNA. The [Ch][Lac] IL was able to preserve the double-helicoidal structure of the nucleic acid during its long-term storage of 6 months at room temperature (Vijayaraghavan et al., 2010). Moreover, Dinis et al. (2020) investigated the use of aqueous solutions of ILs, formed by the combination of cholinium, tetrabutylammonium, tetrabutylphosphonium, and 1-ethyl-3-methylimidazolium cations with bromide, chloride, dihydrogen phosphate, acetate, and glycolate anions, as preservation media for double-stranded DNA (dsDNA). Cholinium-based ILs shown to be most effective in preserving the structure of the dsDNA majorly due to the electrostatic interactions established between the cholinium cation and the phosphate groups of dsDNA, which were determined by ^31^P NMR spectroscopy. Moreover, the denaturation of dsDNA mainly arose with ILs comprising more hydrophobic cations that are capable to establish dispersive interactions with the nucleobases environment of dsDNA. It was also demonstrated that, in contrast to the IL cation, the anion component of the IL has a lower effect in the interaction with dsDNA.

Pedro et al. (2018) evaluated the potential of several self-buffering cholinium-based Good’s buffer ILs (GBILs) at 20 and 50% (w/w) as structural preservation media of recombinant small RNAs (sRNAs) from *E. coli* DH5α strain comprising the therapeutic pre-miR-29. It was found that GBILs were able to increase the stability of the nucleic acid for at least up to 30 days at room temperature and 4°C, allowing to increment the overall shelf-life of the molecule. The ILs [Ch][HEPES] and [Ch][MES] were identified as the best ones in increasing the integrity and stability of sRNAs (Pedro et al., 2018). Their thermal stability was significantly improved, being observed an increase of 14°C in the respective biopolymer melting point. Moreover, no cytotoxicity was observed in two human cell lines for the RNA formulated in ILs solutions at 20% (w/w). This work revealed the potential of using aqueous solutions of GB-ILs as stabilizing and preservation media for recombinant sRNAs at room temperature, avoiding the employment of conventional freezing methodologies during storage. In the same way, Quental et al. (2019) confirmed the RNA stability and integrity for 15 days in aqueous solutions of ILs comprising anions derived from amino acids (AA-ILs). Furthermore, the authors propose an integrated extraction-preservation approach for RNA, in which after extraction-purification of RNA to the IL-rich phase, it can be preserved in that same phase/solution up to its use.

Byrne et al. (2012) studied the effect of ILs as solvents for the solubilisation and stabilization of Tobacco Mosaic Virus (TMV). It was demonstrated that when TMV was formulated with Protic ILs (PILs), the IL enabled to increase its shelf-life to 4 months without significant losses on its tertiary structure (Byrne et al., 2012). In contrast, when TMV was formulated with a conventional buffer solution, its degradation occurred after 3 weeks (Byrne et al., 2012). Therefore, ILs can be seen as promising stabilizing agents for TMV, being this observation highly important for the development of safe viral formulations with therapeutic activity. Also, Lin et al. (2019) studied the effect of biocompatible cholinium-based ILs on the stability of foot-and-mouth disease virus (iFMDV) particles. It was shown that [Ch][Cl] and [Ch][SO_4_] were able to enhance the thermal and long-term stability of the virus, inhibiting its dissociation (Lin et al., 2019). The results obtained in this work showed that if a proper selection of the anion component of the IL is carried out, cholinium-based ILs can be seen as promising stabilizing agents for iFMDV particles, enabling the development of more stable formulations of these products.

In summary, the described works demonstrated the outstanding performance of ILs to improve and/or maintain the stability of relevant biopharmaceuticals, opening the door for their use in formulation development.

## SWOT analysis of Ionic liquid-based approaches in biomanufacturing

**FIGURE 8:**
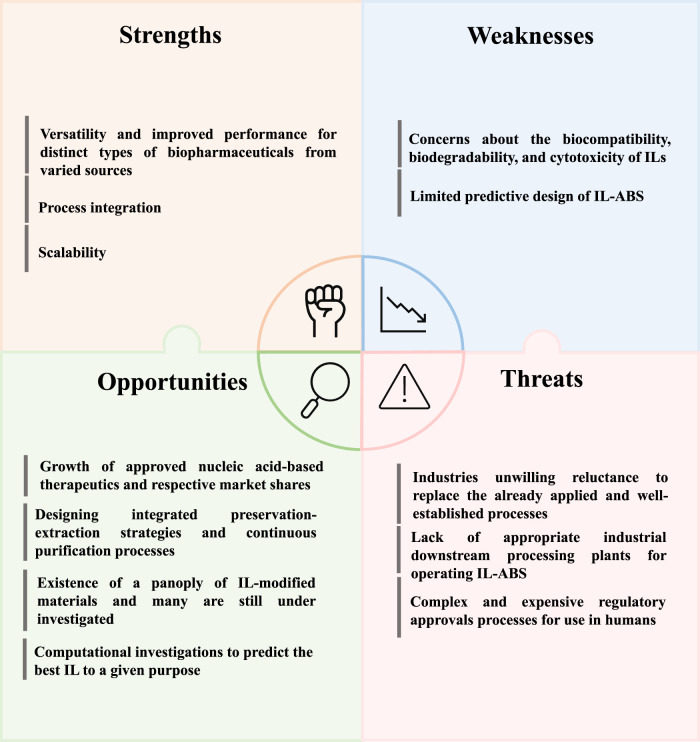
SWOT analysis emphasizing the major strengths, weaknesses, opportunities, and threats of the application of IL-based approaches for the downstream processing and formulation of biopharmaceuticals.

This review has given the reader an overview of the potential of IL-based strategies in biomanufacturing namely, to improve the performance of the downstream processing and formulation development stages of biopharmaceuticals. Despite the significant research advances made in the last few decades, there is still a long road to walk until ILs are widely approved by regulatory agencies and employed at an industrial scale. As summary of the previous discussion, a SWOT analysis (strengths, weaknesses, opportunities, and threats) analysis is presented in Figure 8.

It is important to highlight that a comprehensive SWOT analysis has been previously made by Soares et al. (2015) concerning the use of ABS for the partitioning of biomolecules. The effectiveness, simplicity, biocompatibility, selectivity, integrability, scalability, as well as the possibility of being applied in a continuous operation mode, were referred as ABS major strengths for biomolecule purification. On the opposite, as weaknesses, there is a lack of knowledge concerning the installation, validation, and operation of ABS-based technologies, and a limited predictive design. Regarding IL-based strategies in the downstream processing of biopharmaceuticals, including not only IL-ABS but also SILs, there are three major strengths: i) their versatility and improved/tailored separation performance for different types of biopharmaceuticals from a myriad of sources; ii) the possibility of process integration; iii) and scalability. Due to the outstanding properties presented by ILs, that are transferred to SILs, it is possible to design numerous combinations of ionic components to improve the performance of the envisioned application (Castro et al., 2019; Kianfar and Mafi, 2020; Curreri et al., 2021; Jadhav et al., 2021; Bernardo et al., 2022). However, a proper selection of the cation and anion should consider the envisaged physicochemical properties of the final formulation, the formulation biocompatibility, and the biological activity of the biomolecules. Running the production and purification processes in a continuous mode during biomanufacturing, by integrating the production and purification stages and/or the different purification steps, as shown with the use of ABS in CPC (Soares et al., 2021), use of SILs in preparative liquid chromatography (Neves et al., 2020), and integrated extraction-preservation approaches (Quental et al., 2019), could contribute to further improve the cost-effectiveness and potential application at large-scale of these processes.

Notwithstanding the benefits previously mentioned, there are still two major weaknesses limiting the broad application of IL-based approaches in biomanufacturing (Tavares et al., 2019): the concerns regarding the biocompatibility, biodegradability, and cytotoxicity of ILs, and (Kesik‐Brodacka, 2018) the limited predictive design of IL-ABS. Furthermore, before their use in the biopharmaceuticals field can become a reality, a long path including *in vivo* preclinical studies and long and expensive human clinical trials to assess safety and efficacy is still needed. Since the evaluation of ILs cytotoxicity and biocompatibility is mandatory when envisioning their application in the biopharmaceutical field (Lin et al., 2021), ILs comprising cations and anions from a natural source and/or already approved as safe ingredients by regulatory entities should be preferably selected.

Several opportunities could be pointed out for IL-based approaches in biomanufacturing, among them (Tavares et al., 2019): the fact that the number of approved nucleic-acid-based therapeutics and respective market shares is growing, in part due to the emergence of the COVID-19 mRNA vaccines (Kesik‐Brodacka, 2018); the possibility of designing integrated preservation-extraction strategies and continuous purification processes (O’Flaherty et al., 2020); the existence of a panoply of ILs and IL-modified materials allowing to tailor separation performance; and (Ferreira et al., 2016) the possibility of performing computational investigations to predict the best IL to a given purpose. IL-based strategies still cope with some threats that limit their use in bioprocesses, namely the unwillingness of some industries to substitute their current and well-established processes, the lack of appropriate industrial downstream processing plants for operating IL-ABS, and the complex and expensive regulatory approval processes required for the use of biopharmaceuticals in humans.

## Conclusions and future perspectives in the field

ILs and IL-modified materials can be considered highly promising tools to improve the downstream processing and formulation approaches of biopharmaceuticals. Several IL-based approaches, most of them relying on IL-ABS and SILs, have been proposed confirming their outstanding performance to enhance the recovery yields and purification efficiency of a myriad of biomolecules, including therapeutic enzymes, antibodies, nucleic acids, viruses, and interferons. The attractiveness of using IL-based approaches in the biopharmaceutical field is due to their remarkable properties, particularly versatility and “designer” nature, allowing them to modulate the final properties and outcomes of a specific process/application and therefore, to surpass the challenges related to the lack of robust, selective, and easy to scale-up approaches for the recovery, purification, and formulation of biopharmaceuticals.

In the downstream processing, IL-based ABS/ATPMS/TPP and SILs have shown the ability to improve the purity and yield of a myriad of biopharmaceuticals. Research showing the possibility of performing biomolecules’ purification in continuous mode with ABS and CPC, and the identification of ILs as multimodal ligands in preparative liquid chromatography, seems particularly relevant. It is also important to emphasize the capacity of ILs to be recovered and reused, contributing to the development of more sustainable and low-cost processes. In the formulation field, ILs have been shown to have high potential to improve the thermal and chemical stability of several therapeutic compounds, and to inhibit their aggregation, denaturation, enzymatic degradation, and fragmentation.

Other factors, such as the safety profile and costs, also need to be adequately assessed before considering market introduction. However, ILs and SILs-based processes represent good alternatives when aiming to reduce biomanufacturing-associated environmental, economic and health burdens. Although there is still a long path ahead, the research conducted so far and here reviewed shows the remarkable potential of IL-based approaches in the biopharmaceutical field, challenging researchers to go above and beyond.
