# Corrigendum: Ionic-liquid-based approaches to improve biopharmaceuticals downstream processing and formulation

**DOI:** 10.3389/fbioe.2023.1213921

**Published:** 2023-05-09

**Authors:** Catarina Almeida, Augusto Q. Pedro, Ana P. M. Tavares, Márcia C. Neves, Mara G. Freire

**Affiliations:** CICECO-Aveiro Institute of Materials, Department of Chemistry, University of Aveiro, Aveiro, Portugal

**Keywords:** biopharmaceuticals, ionic liquids, biomanufacturing, downstream processing, purification platforms, formulation, ionic liquid-based approaches, biopharmaceuticals administration

In the published article, there was an error in the **Funding** statement. The reference for the project AntYmicrob was displayed as “CENTRO -01-0247-FEDER-181219” and should be “CENTRO -01-0145-FEDER-181219.”

The correct **Funding** statement appears below.

“This work is financed by Portugal 2020 through European Regional Development Fund (ERDF) in the frame of CENTRO2020 in the scope of the project AntYmicrob, CENTRO -01-0145-FEDER-181219 and in the scope of the project CICECO—Aveiro Institute of Materials, UIDB/50011/2020 and UIDP/50011/2020 and LA/P/0006/2020, financed by national funds through the FCT/MEC (PIDDAC). This work was additionally developed within the scope of the EIC-Pathfinder YSCRIPT project with reference 101047214, supported by the budgets of the Horizon Europe Program.”

The authors apologize for this error and state that this does not change the scientific conclusions of the article in any way. The original article has been updated.

